# Spontaneous pleural aspergillosis in an immunocompetent young adult treated with minimally invasive surgery

**DOI:** 10.1016/j.rmcr.2023.101869

**Published:** 2023-05-11

**Authors:** Hiromi Ichikawa, Ryoichiro Doi, Keitaro Matsumoto, Koichi Tomoshige, Masataka Hirabaru, Ryusuke Machino, Tomohiro Obata, Satoshi Mizoguchi, Junji Irie, Tomoshi Tsuchiya, Takeshi Nagayasu

**Affiliations:** a Department of Surgical Oncology, Nagasaki University Graduate School of Biomedical Sciences, Sakamoto 1-7-1, Nagasaki, 852-8102, Japan; b Department of Respiratory Surgery, Nagasaki Harbor Medical Center, Shinchi-machi 6-39, Nagasaki, 850-0842, Japan; c Department of Pathology, Nagasaki Harbor Medical Center, Shinchi-machi 6-39, Nagasaki, 850-0842, Japan; d Department of Thoracic General Surgery, Toyama University Hospital, Sugitani 2630, Toyama, 930-0194, Japan

**Keywords:** Pleural aspergillosis, Aspergillus empyema, Spontaneous pneumothorax, Decortication, Cavernostomy, Minimally invasive surgery

## Abstract

Spontaneous cases of pleural aspergillosis in healthy adults are rare, and the optimal therapeutic approach has not been established. Here we report a rare case of spontaneous pleural aspergillosis in an otherwise healthy young adult. Two-stage surgery with decortication and cavernostomy, followed by systemic antifungal therapy, finally resulted in a successful resolution of his empyema without any serious complications. In young patients with good pulmonary compliance, less invasive procedures, such as thoracoscopic decortication and/or carvernotomy, is a potential treatment option.

## List of abbreviations

VRCZ: voriconazole

## 1 Introduction

Pleural aspergillosis is defined as direct Aspergillus infection of the pleura or pleural space and is a rare entity among the spectrum of Aspergillus infections [1]. Pleural aspergillosis is more commonly associated with the presence of a bronchopleural fistula, the formation of which is associated with previous pulmonary tuberculosis [2] and thoracic surgery [3]. Colonization during procedures and/or direct communication between the airways and pleural space may increase the risk of direct inoculation, with Aspergillus dissemination into the pleural space. However, spontaneous cases of pleural aspergillosis in healthy adults are extremely rare, and only a few cases have been reported to date [[4], [5], [6], [7], [8]].

Thoracic fungal infections are associated with a high risk of mortality [1] and, mostly due to its rarity and various underlying pulmonary diseases, the optimal therapeutic approach for pleural aspergillosis has not been established. Therefore, we need to manage each case of pleural aspergillosis with respect to the cause and the condition of the patient. Here, we report the rare case of a previously healthy 22-year-old male patient who developed pleural aspergillosis in the entire left pleural cavity as a complication of a pre-existing underlying chronic pneumothorax, and discuss possible therapeutic options in this context.

## 2 Case presentation

**Fig. 1:**
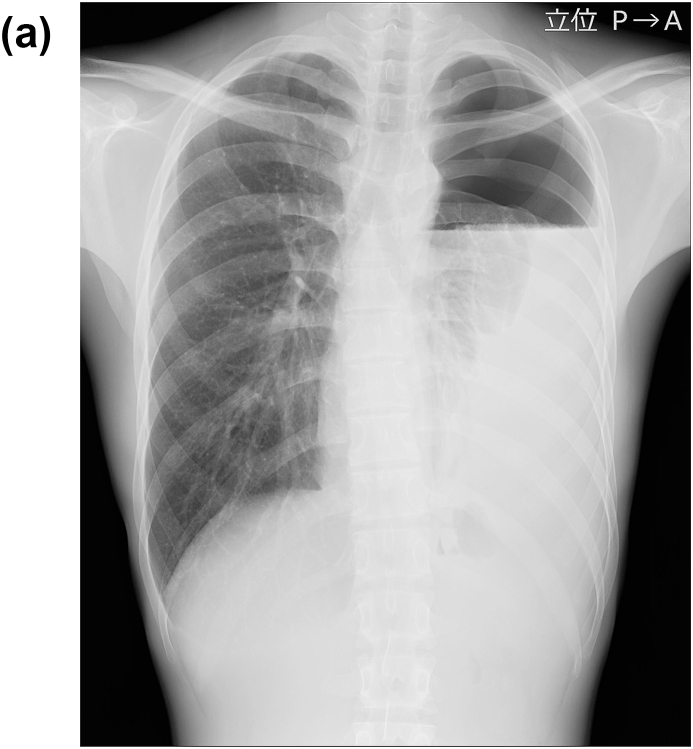

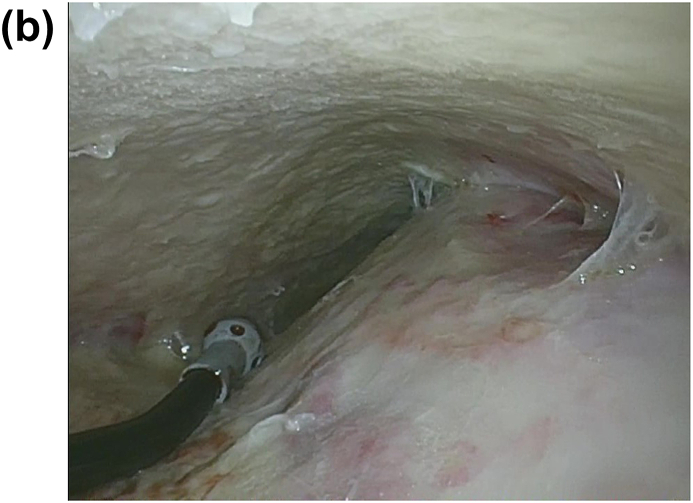

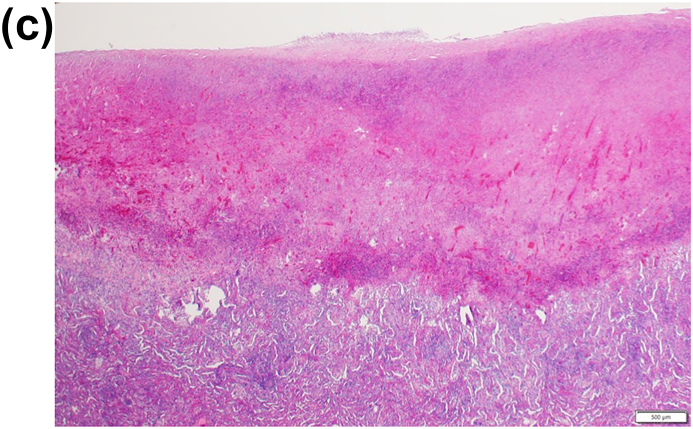

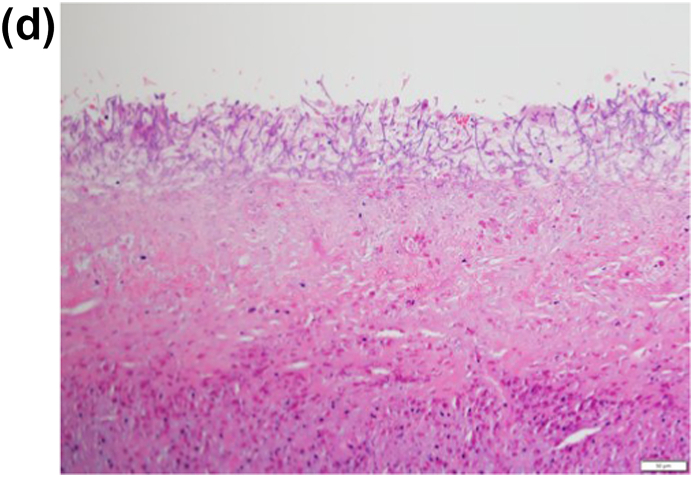
(A) Chest radiography revealed pneumohydrothorax in the left thoracic space. (B) Thoracoscopic view of the infected left pleural space. *Aspergillus fumigatus* was cultured from the resected pleura.C,D) The microscopy image of the excised specimen showed that the lesion was confined to the visceral pleura and consisted of necrotic nests and inflammatory cell nests, and the organisms of Aspergillus were located within the visceral pleura, whereas not in the lung parenchyma (hematoxylin and eosin stain, 20x (C) and 400× (D) magnification).

**Fig. 2:**
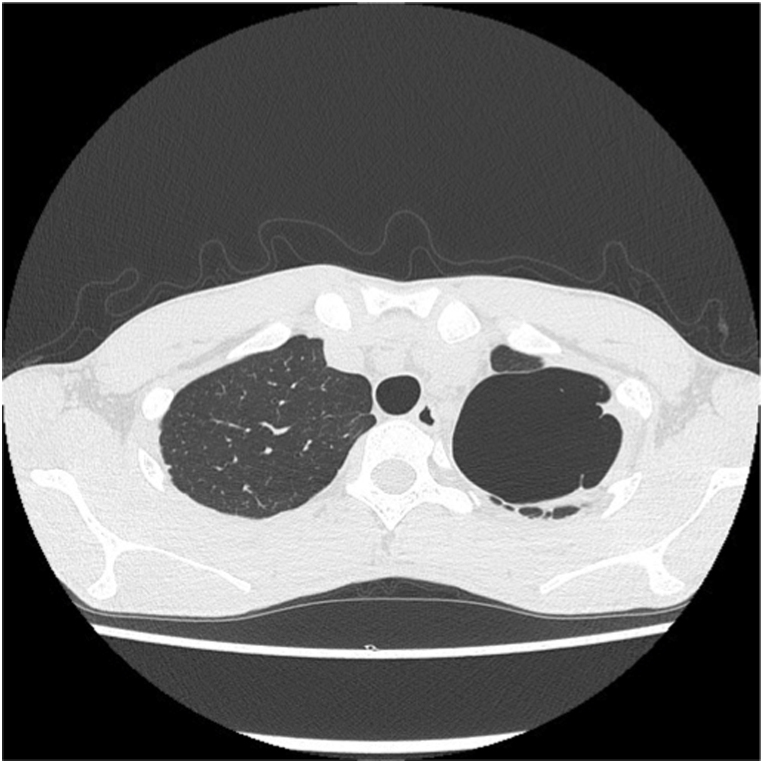
One month after the decortication, re-infection with Aspergillus occurred and persistent air leak developed. Chest computed tomography revealed parietal pleural thickening in the apical thoracic cavity.

A 22-year-old male patient had been occasionally experiencing left chest pain and dyspnea for several years, but the symptoms had resolved spontaneously, and he had no history of medical examination. He presented to his home physician with persistent complaints of respiratory distress and high-grade fever for one month after the onset of left chest pain and dyspnea, and was found to have a pneumohydrothorax on chest radiography (Fig. 1A). For the failure of conservative treatment, he underwent thoracoscopic decortication at a local hospital (Fig. 1B). The apical part of the left upper lung, which could not be peeled off, was resected. A pathological finding of Aspergillus organisms in the pleura (Fig. 1C and D), and a serological positiveness of Aspergillus antibodies led to the diagnosis of spontaneous pneumothorax and Aspergillus empyema. The patient had no history of pulmonary aspergillosis, and no organisms were present in the lung parenchyma in the lung resection specimen. Laboratory and physical findings were negative for the presence of any disease that could cause opportunistic infections, and there was no history of any other noteworthy illness since birth and no family history of immunodeficiency diseases. It was strongly inferred the patient had developed a chronic pneumothorax cavity as a result of repeated pneumothoraces, although there was no history of the other diseases that could form cavitary lesions, such as tuberculosis. Just before the onset of symptoms, his apartment had been closed for an extended period for the summer vacation, and upon his return, he discovered that his place was heavily infested with mold due to the high summer temperatures and humidity. It was presumed that the patient developed the current pneumothorax in this moldy environment, resulting in the dissemination and infection of Aspergillus in the previously existing chronic pneumothorax cavity through the bronchopleural space. The postoperative course after the decortication and the wedge resection was good, and he was discharged home on the eighth postoperative day. However, one month after the operation, re-infection occurred and persistent air leak developed, which prompted admission to our hospital. Chest computed tomography (CT) revealed a large apical pleural space in the left without a fungus ball, and an irregularly thickened pleura in the upper thoracic cavity, suggesting the infection of the pleura and air space (Fig. 2). Pleural fluid culture detected *Aspergillus fumigatus*, and serum Aspergillus antibodies were again positive. Other findings on CT included a very mildly infiltrated left upper lung parenchyma with intrapulmonary infection at the bottom of the cavity. The patient had no evidence of intrapulmonary infection on imaging or histology at the time of the initial surgery, suggesting that the fungus in the pleural and pneumoperitoneum had spread to the lungs after the previous surgery; hence, the patient was diagnosed with apical pleural aspergillosis because the main body of infection was located in the pleura and airspace.

It was determined that surgical intervention for the infection site in the pleura was necessary for curative treatment. Since the intrapulmonary infection was controlled with antifungal medication, lobectomy with extensive muscle filling was considered an excessive intervention. Because the dead space was confined to the apex of the lung, we decided to perform cavernostomy and minimal dead space filling as the first-line procedure.

**Fig. 3:**
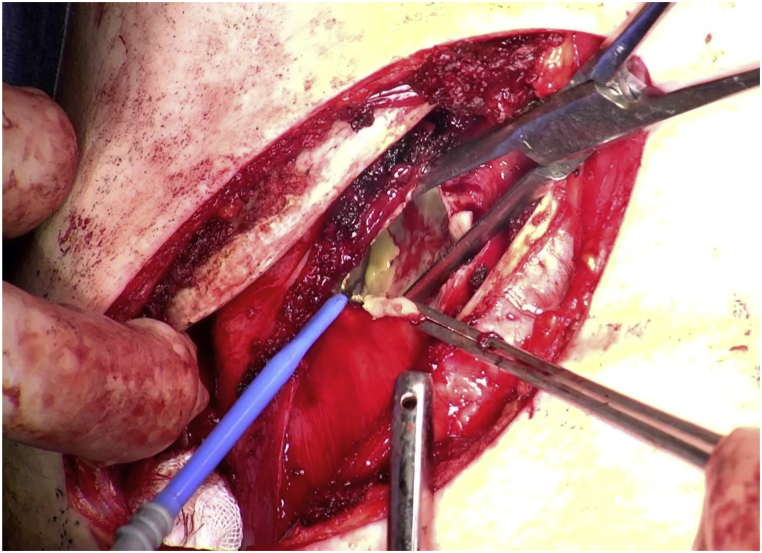
Cavernostomy of the apical Aspergillus cavity was performed via the intercostal space between the 2nd rib and the 3rd rib with extraperiosteal air-plombage.

A 12-cm anterolateral incision was made in the third intercostal space, and cavernostomy was performed on the Aspergillus cavity, providing a clear view from apex to bottom (Fig. 3). The thickened parietal pleura, forming the cavity wall, was deemed unresectable, while the thickened lung parenchyma at the cavity bottom maintained compliance. To control infection, the pyothorax cavity was scraped with a sharp curette to remove Aspergillus fragments, and the alveolar fistula was closed by suturing the pleura as a pledget. The cavity volume was estimated at 90 mL. The extraperiosteal air-plombage method (Kinchu method) was used to minimize cavity volume. Intercostal muscle and partial serratus anterior muscle transposition flaps, attached to the second and fourth ribs, were guided into the cavity. Two thoracic drains were placed in the cavity, one outside the pleural tent, and the wound was closed.

**Fig. 4:**
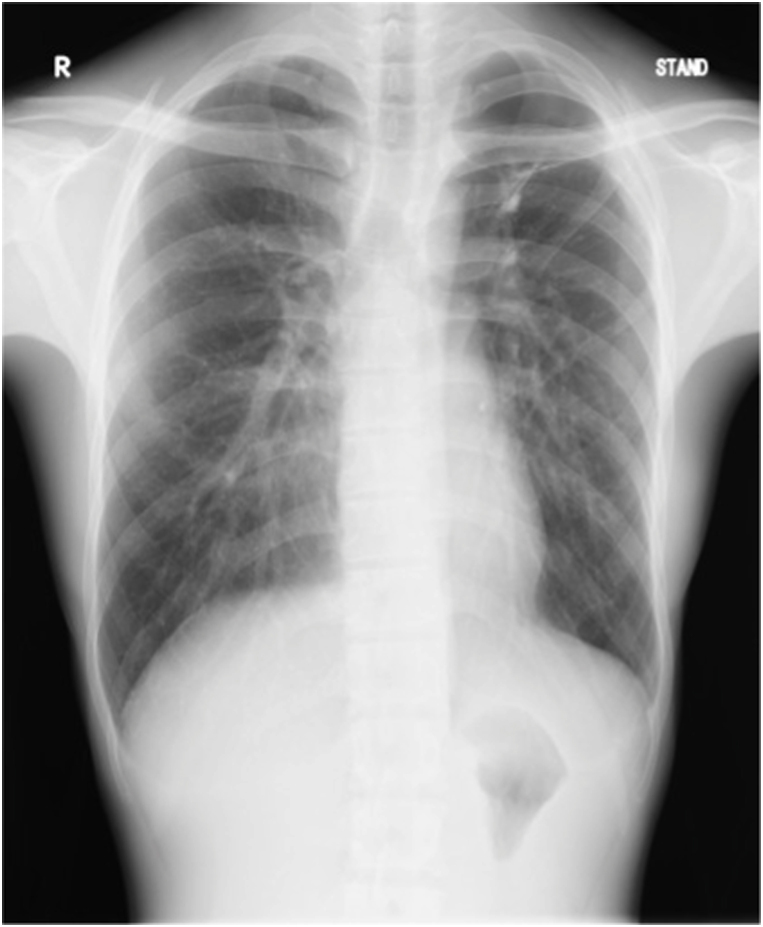
Chest radiography one year after the operation showed full reexpansion of the left lung.

Postoperatively, the thoracic drain was maintained for three weeks while the patient underwent intravenous voriconazole (VRCZ) therapy (4 mg/kg every 12 hours for 4 weeks), which was eventually transitioned to oral VRCZ (400 mg/day) for approximately a year. One year after the surgery, microbiological results remained negative for the organism. The patient had good lung reexpansion, with full extension into the bony thorax (Fig. 4). With regard to scapular dysfunction, the patient developed slight limitation in movement when raising the left upper limb straight toward the head and putting it on the ear, but there has been no other movement disorder of the upper limb or scapula, and no difficulty in daily life. There is no thoracic deformity or disfigurement.

## 3 Discussion

Pleural aspergillosis is a rare form of Aspergillus infection that is not always associated with pulmonary infection [9]. Curiously, it can occur in immunocompetent patients, even in healthy young adults [5]. Additionally, most cases of pleural aspergillosis are secondary to tuberculosis or thoracic surgery/interventions. Furthermore, spontaneous cases without underlying pulmonary diseases are extremely rare. The background of each case of pleural aspergillosis is different. Therefore, although there are guidelines for pulmonary aspergillosis, there are no clearly defined therapeutic pathways for pleural aspergillosis [10]. Some cases have been successfully treated with conventional systemic antifungal therapy and thoracic drainage without pulmonary resection [11]. Surgical intervention for pleural aspergillosis is a significant undertaking and has associated technical difficulties because the typical patient population may have had previous thoracic surgery. A combination of medical therapy and surgical intervention likely provides the best outcomes [12].

Surgery for pleural aspergillosis follows the treatment principles of empyema. The key principles of surgery for chronic empyema are infection control by debridement, drainage, and closure of the fistula and cavity with dead space filling. In general, surgical treatment of empyema usually involves either decortication or thoracoplasty, which can be challenging in this population. For reference, the most recommended procedure for pulmonary aspergilloma is lobectomy and decortication to achieve complete resection of the lesion or control of the disease [13,14]. Additionally, if the residual space after lobectomy is large, some patients with multi-cavity disease may require thoracoplasty with simultaneous muscle transposition flap. Alternatively, cavernosotomy may be performed in patients with poor lung function or nutritional status [13]. The results of cavernostomy have been surprisingly good, with no surgery-related deaths or serious complications, and long-term results are comparable to those of anatomical resection [13].

Reported cases of spontaneous pleural aspergillosis with pneumothorax were treated with relatively less invasive surgical procedures, such as surgical debridement [5] or right middle lobectomy alone [8]. One-stage surgery was performed for the localized empyema cavity in previous cases. In our case, thoracoscopic decortication and apical pulmonary wedge resection as first-stage surgery was performed for empyema in the left pleural cavity, and this procedure enabled complete reexpansion of the left lower lung. However, apical pleural aspergillosis developed in the apical space. Thoracic drainage and systemic antifungal therapy could not cure the apical pleural aspergillosis due to persistent air leakage via an alveolar fistula. At this time, left upper lobectomy and decortication with muscle filling were considered as potential curative treatments. However, these options could lead to functional and cosmetic disabilities, impacting the patient's quality of life. Since the infected foci in the left upper lobe improved with preoperative antifungal medication, and the main infection site was in the pleura and dead space, we opted for cavernostomy and minimal dead space filling as the first-line procedure. This surgical technique does not impair the patient's respiratory function and preserves the latissimus dorsi and pectoralis major muscles. Cavernostomy as the second surgery finally resolved the apical pleural aspergillosis. The preserved residual lung parenchyma was able to fill the dead space in the apical cavity of the left thoracic cage. Good pulmonary compliance was an important factor that resulted in the favorable clinical outcome, preserving pulmonary function, avoiding chest wall deformity, and minimizing upper limb dysfunction.

## 4 Conclusion

In otherwise healthy patients with no underlying pulmonary disease, surgical procedures such as lobectomy and thoracoplasty for pleural aspergillosis can be overly invasive and can significantly compromise the patient's quality of life. Decortication and/or cavernostomy, less invasive surgical approaches, would be effective and sufficient as the definitive surgical procedures for such cases and may be emerging options that should be considered.

## Funding sources

This research did not receive any specific grant from funding agencies in the public, commercial, or non-profit sectors.

## Author's contributions

All the authors contributed to the treatment of the patient.

## Patient consent for publication

Informed consent was obtained from the patient.

## Ethical statement

All medical procedures performed adhered to the tenets of the Declaration of Helsinki.

## Declaration of competing interest

The authors have no conflicts of interest to disclose.
